# Early-onset bibasilar emphysema in a patient with PLCG2-related immune dysregulation

**DOI:** 10.1016/j.rmcr.2025.102308

**Published:** 2025-10-18

**Authors:** Annika Sundlof, Justin Nathan, Glenn S. Gerhard, Daniel Salerno

**Affiliations:** Temple University Hospital, 3401 North Broad Street, Philadelphia, PA 19140, United States

**Keywords:** COPD, Emphysema, PLCG2, PLAID, APLAID

## Abstract

Mutations in the PLCG2 gene, which encodes an enzyme in the intracellular signaling pathway of B lymphocytes, result in a spectrum of conditions involving frequent infections, antibody deficiencies, immune dysregulation, and cutaneous manifestations. These conditions are coined APLAID. We describe a case of early-onset bibasilar emphysema in an individual with frequent respiratory infections and immunoglobulin deficiencies, found to have a PLCG2 mutation on genetic testing. This case is unusual because the patient has bibasilar emphysema that is unrelated to alpha-1 antitrypsin deficiency, and because COPD is not a previously described manifestation of APLAID. The mechanism causing this phenotype is unclear; it is possible that frequent infections lead to lung parenchymal damage and emphysema, or perhaps there is an association between autoimmunity and emphysema. With this case, we expand the spectrum of PLCG2-related phenotypes, highlight the utility of genetic testing, and review the literature on possible treatment options including IVIG and corticosteroids.

## Highlights

• Unusual case of bibasilar emphysema without alpha-1 antitrypsin deficiency.
• Early-onset emphysema as a novel phenotype of PLCG2 mutation.
• IVIG, prednisone, and other potential treatment options for PLCG2-related conditions.

## Glossary:

COPD: chronic obstructive pulmonary disease
PLAID: PLCG2-related antibody deficiency and immune dysregulation
APLAID: autoinflammation and PLCG2-related antibody deficiency and immune dysregulation
PLCG2: phospholipase C gamma 2

## 1 Background

Phospholipase Cγ2 (PLCG2) is an enzyme that amplifies intracellular responses to surface receptor activation in B lymphocytes by generating secondary messengers (DAG and IP3). These messengers increase intracellular calcium levels, reinforcing a positive feedback loop that enhances PLCγ2 activation and therefore the response to extracellular signals [1]. Given B lymphocytes' critical role in humoral immunity, PLCγ2 is fundamental to immune system function.

Since 2012, PLCG2 variants have been linked to a spectrum of immune-related conditions, including autoinflammatory disorders, immune deficiencies, and cutaneous manifestations. Genomic deletions in PLCG2 cause PLAID (PLCG2-related antibody deficiency and immune dysregulation), characterized by cold urticaria, immunoglobulin deficiencies, recurrent infections, atopy, and autoimmunity [2]. Missense mutations result in APLAID (autoinflammation and PLCG2-related antibody deficiency and immune dysregulation), which may present with bullous skin lesions, bronchiolitis, enterocolitis, ocular inflammation, and recurrent infections [3,4]. However, clinical presentations vary widely among affected individuals, with most patients exhibiting immunoglobulin deficiency, recurrent infections, and autoimmunity to varying degrees [5].

The missense variant Met1141Lys has been identified in three previously reported cases across two families. Two individuals (a mother and child) exhibited APLAID-like symptoms, including autoinflammation and recurrent infections, and the mother was noted to develop COPD. The third patient, diagnosed with pediatric CVID, experienced recurrent viral and bacterial infections; his father died prematurely of emphysema. Functional studies demonstrated that Met1141Lys is a gain-of-function mutation that enhances calcium flux in B cells [6], which is theorized to result in the immune dysregulation noted in those cases.

Here, we present a case of a non-smoking patient with early-onset emphysema and immunoglobulin deficiency, subsequently found to carry the PLCG2 Met1141Lys variant. While emphysema has been reported in some cases of APLAID, its prominence as the primary clinical manifestation in this patient is unusual. This case expands the spectrum of PLCG2-related phenotypes.

## 2 Case presentation

The patient is a non-smoking female, aged mid-forties, with a history of early-onset emphysema, immunoglobulin deficiency, and recurrent respiratory infections since childhood. She was found to harbor the PLCG2 Met1141Lys variant.

**Fig. 1:**
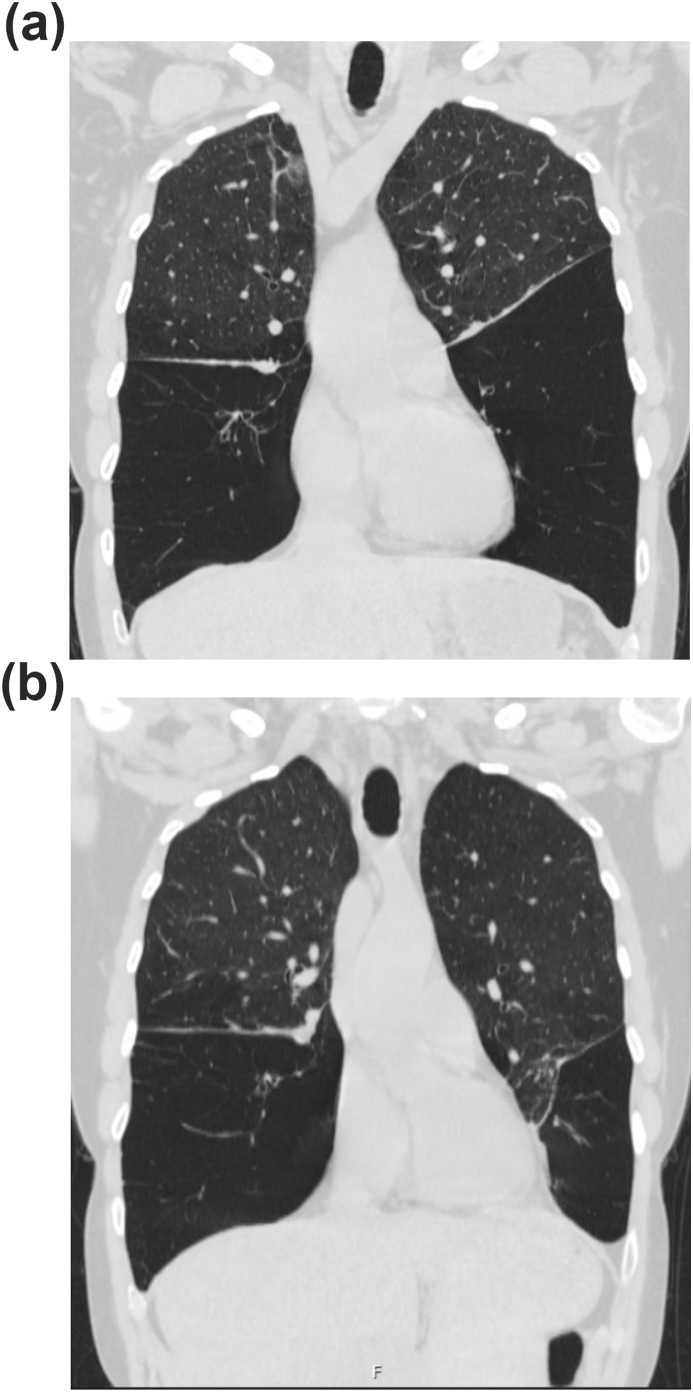
CT imaging illustrates severe destructive emphysema with lower lobe predominance. Panel A shows a coronal CT prior to left lower lobe endobronchial valve placement, and Panel B shows the same view after endobronchial valve placement.

The patient experienced exertional dyspnea beginning in her mid-20s and was later diagnosed with severe COPD at age 38. CT imaging revealed emphysema with lower lobe predominance (Fig. 1A). Pulmonary function tests demonstrated GOLD grade 3 airflow obstruction, air trapping, and hyperinflation. She denied smoking, secondhand smoke exposure, or environmental fumes exposure. Notably, her father also died of COPD. Testing for alpha-1 antitrypsin deficiency and cystic fibrosis was negative, prompting genetic testing, which revealed the PLCG2 Met1141Lys variant of uncertain significance. During this diagnostic workup, she responded to triple therapy inhalers and low-dose prednisone, and she underwent endobronchial lung volume reduction (Fig. 1B) with improvement in her dyspnea.

Further evaluation revealed a history of recurrent respiratory infections, including frequent colds in childhood and bronchitis up to four times per year in adulthood, often requiring antibiotics and steroids. She also experienced recurrent sinusitis. Past sputum cultures grew Hemophilus influenzae and methicillin-sensitive Staphylococcus aureus. Laboratory testing demonstrated IgA and IgG subclass 1 deficiency (Table 1). Despite referral to infectious diseases and attempts to initiate IVIG therapy, socioeconomic and insurance barriers delayed treatment.

**Table 1:** Deficiency in IgA and IgG subclass 1.

Immunoglobulin	Result	Reference range
IgE	7	<100 IU/mL
IgA	***74 (L)***	83–407 mg/dL
IgG	836	680–1445 mg/dL
IgM	146	34–214 mg/dL
**IgG Subclasses**		
IgG Subclass 1	***375 (L)***	382–929 mg/dL
IgG Subclass 2	243	242–700 mg/dL
IgG Subclass 3	***>220 (H)***	22–176 mg/dL

## 3 Discussion

This case aligns with previously described PLAID/APLAID presentations in terms of immunoglobulin deficiency and recurrent infections, but our patient's clinical course is unusual in that severe emphysema is the predominant feature. Lower lobe-predominant emphysema without alpha-1 antitrypsin deficiency is particularly notable, broadening the known spectrum of PLCG2-related disorders.

In a cohort of 76 patients with PLCG2 variants, emphysema was not reported as a phenotype of PLAID/APLAID disorders [5]. However, in the only other case series describing Met1141Lys, one patient developed COPD, and another patient's father died of emphysema [6]. Whether this association is incidental or suggests a direct link between Met1141Lys and emphysema remains unclear.

Mechanistically, PLCG2 mutations could contribute to emphysema through recurrent infections, leading to chronic inflammation and lung parenchymal damage. This aligns with findings that primary immunodeficiency may be an underrecognized cause of COPD [7]. Alternatively, PLCG2 mutations may predispose patients to autoimmunity-driven lung injury, akin to proposed autoimmune mechanisms in COPD, wherein immune cells target alveolar structures, triggering progressive destruction [[10], [11]]. Although our patient lacks a formal autoimmune diagnosis, this remains an area for further investigation.

Current management for this patient includes triple therapy inhalers and low-dose prednisone. Endobronchial lung volume reduction was also effective for the patient's dyspnea due to severe emphysema. IVIG therapy remains a potential intervention for her immunoglobulin deficiency and recurrent infections, though the difficulty in initiating this treatment illustrates the importance of social determinants of health in access to potentially beneficial care. Notably, IVIG has been shown to reduce exacerbations in COPD patients with antibody deficiencies [8]. Case reports suggest that corticosteroids and IVIG may benefit PLCG2-related conditions, whereas IL-1 inhibitors yield variable responses, and TNF inhibitors appear ineffective [3,4]. Emerging research also suggests G-CSF as a driver of APLAID inflammation, highlighting potential therapeutic targets [9]. Overall, no consensus treatment for PLCG2-associated conditions exists.

This case underscores the heterogeneity of PLCG2-related diseases, particularly the Met1141Lys variant. It also emphasizes the value of genetic testing in early-onset emphysema with an unclear etiology, as such findings may influence disease management and family screening.

## CRediT authorship contribution statement

**Annika Sundlof:** Writing – original draft, Writing – review & editing. **Justin Nathan:** Writing – original draft, Writing – review & editing. **Glenn S. Gerhard:** Writing – original draft, Writing – review & editing. **Daniel Salerno:** Conceptualization, Writing – original draft, Writing – review & editing.

## Declaration of competing interest

The authors declare that they have no known competing financial interests or personal relationships that could have appeared to influence the work reported in this paper.
